# Receptor-like Kinases in Crop Breeding: Potentials and Challenges

**DOI:** 10.3390/plants15213363

**Published:** 2026-10-30

**Authors:** Meizhen Li, Chunrong Ye, Minghui Lv, Jia Li

**Affiliations:** 1 Innovation Center for Cell Signal Transduction and Synthetic Biology, School of Life Sciences, Guangzhou University, Guangzhou 510006, China; 2 Guangzhou University Branch Center of the State Key Laboratory of Non-Food Biomass Energy Technology, School of Life Sciences, Guangzhou University, Guangzhou 510006, China; 3 Guangdong Provincial Key Laboratory of Plant Adaptation and Molecular Design, School of Life Sciences, Guangzhou University, Guangzhou 510006, China; 4 Ministry of Education Key Laboratory of Cell Activities and Stress Adaptations, School of Life Sciences, Lanzhou University, Lanzhou 730000, China

**Keywords:** receptor-like kinase, breeding, yield, resistance, tolerance

## Abstract

Plant receptor-like kinases (RLKs) constitute one of the largest and most ancient protein families in land plants. Localized on the plasma membrane, they perceive a diverse array of extracellular signals and coordinate intracellular responses that are crucial for plant growth, development, immunity, and adaptation to environmental changes. Over the past two decades, there has been an explosive expansion of knowledge regarding how RLKs govern virtually every aspect of plant life. Crucially, this knowledge has been translated into practical applications for crop improvement. Here, we summarize the conspicuous triumphs of RLK-mediated strategies in improving plant architecture and yield, enhancing biotic stress resistance against insect herbivores and microbial pathogens, as well as bolstering abiotic stress tolerance to heat, cold, drought, and salinity. We also discuss how RLKs have been utilized as precision molecular targets through genome editing, promoter engineering, and other biotechnological approaches, offering a roadmap for sustainable crop improvement.

## 1. Introduction

Food is a paramount necessity of the people. Due to the growing global population, the demand for both the quantity and quality of food is continuously increasing [1,2]. However, climate change and the reduction in arable land pose new challenges to the productivity and adaptability of food crops [1,2]. As a result, breeders and plant scientists are under pressure to improve existing crop varieties or develop new ones with higher yield and better stress resistance [1,2]. Over the past century, breeding strategies have evolved from traditional cross-breeding and selection to cutting-edge biotechnologies [3,4]. The advent of genome editing, particularly CRISPR-Cas9 technology, has revolutionized trait improvement by allowing targeted modifications at specific genomic loci with unprecedented accuracy and speed [3,4]. Crop breeding is entering a new era of precision design, promising resilient and nutritious crops tailored to meet future agricultural demands [5]. At the molecular level, engineering key regulatory genes that govern plant growth, development, and stress responses holds tremendous potential for accelerating this process. Among such regulatory components, receptor-like kinases (RLKs) have emerged as critical players in mediating environmental signals and intracellular communications [6,7].

## 2. RLKs and Transmembrane Signaling in Plants

RLKs are encoded by a large gene family, representing ~1–2.5% of the coding genes in many plant genomes [8,9]. Plant RLKs belong to the RLK/Pelle superfamily, which also includes the animal Pelle receptor kinases [10]. Phylogenetic analyses suggest that RLK/Pelle kinases share a monophyletic origin with the well-characterized animal receptor tyrosine kinases (RTKs) [10]. Both the RLK/Pelle and RTK families have undergone extensive, lineage-specific expansions, largely driven by tandem duplications and whole-genome duplication events [10,11]. These expansions are believed to be closely linked to the evolution of multicellularity and the increasing demand for complex cell–cell communications [10,11].

Typical plant RLKs are transmembrane proteins composed of an N-terminal extracellular domain (ED), a single-pass transmembrane segment, and a C-terminal intracellular kinase domain (KD) [12]. The KDs are relatively conserved and adopt a canonical bi-lobal kinase architecture. They catalyze the transfer of the γ-phosphate from ATP to downstream protein substrates, thereby modulating their functionality [13]. In contrast, EDs of RLKs differ drastically in size and architecture, conferring specificity for recognizing diverse signaling ligands, such as peptides, carbohydrates, hormones, and pathogen-associated molecules [8]. Based on the ED structure, RLKs are classified into different subfamilies. The largest RLK subfamily, characterized by EDs composed of varying numbers of leucine-rich repeats (LRRs), is also the most extensively studied RLK family to date [8,11]. Other major subfamilies include S-domain RLKs (SRKs), wall-associated RLKs (WAKs), lectin RLKs (LecRLKs), malectin-like RLKs, and cysteine-rich repeat RLKs (CRKs) [8,11]. It is important to note that the plant RLK superfamily also includes a subgroup of receptor-like cytoplasmic kinases (RLCKs) that lack EDs [8,9,10,11,12]. This subgroup, along with the receptor-like proteins (RLPs), a related family that shares structural similarities with RLKs but lacks KDs, falls outside the scope of this review. A key consideration is that an RLP or RLCK alone is unable to mediate complete transmembrane signal transduction from perception of extracellular signals to initiate intracellular cascades.

RLKs typically exert their functions at the cell surface by forming signaling complexes with themselves, other RLKs, and/or RLPs [13,14,15]. In these complexes, the KDs of RLKs are brought into close spatial proximity, facilitating transphosphorylation reactions that lead to their activation [13,14,15]. The activated KDs then phosphorylate downstream effectors such as mitogen-activated protein kinases (MAPKs) or RLCKs [16,17,18]. Various autocrine, paracrine, or endocrine molecules, also known as ligands, can regulate the assembly dynamics of RLK signaling complexes by functioning either as molecular glue or as antagonists [13,15]. Over the past two decades, numerous studies have demonstrated that ligand–RLK-mediated transmembrane signaling plays a fundamental role in regulating cellular communications during plant growth and development, as well in responses to biotic and abiotic stresses [7,19,20,21,22,23,24]. More intriguingly, studies in crop species have provided direct evidence for the importance of ligand–RLK signaling pathways in the regulation of agronomically important traits.

In this review, we discuss the potentials of RLKs as molecular breeding targets for improving crop architecture and yield, enhancing resistance to herbivorous insects and microbial pathogens, and increasing tolerance to unfavorable environmental conditions. We focus specifically on RLKs whose loss- or gain-of-function mutations markedly enhance target traits while causing no or only minor adverse effects. Wherever possible, we highlight specific genes, mutant alleles, molecular mechanisms, and breeding strategies that have either been successfully developed or hold immediate potential for future agricultural applications. We focus mostly on the application value of RLKs in the breeding of staple crops (including rice, wheat, and maize), while also highlighting important findings regarding the breeding applications of RLKs in other crops, such as tomato, soybean, and rapeseed.

## 3. RLKs in Improving Crop Yield

**Figure 1:**
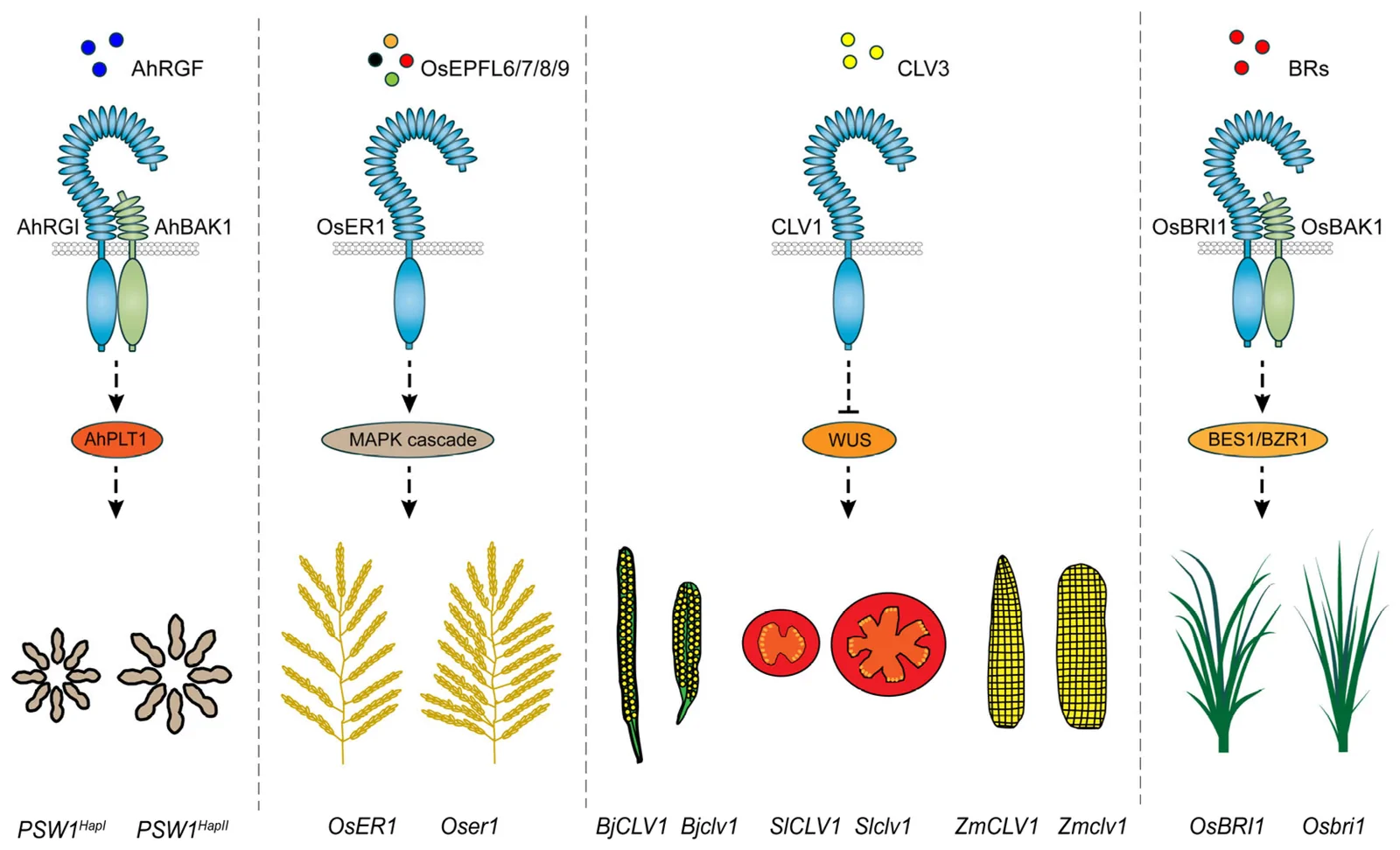
Schematic representation highlighting RLKs as potential molecular targets for improving crop yield and architecture. In peanut, a natural haplotype of *AhRGI* (*PSW1^HapII^*) leads to larger pod size. In rice, repressing ER signaling by knocking out *OsEPFL6/7/9* or *OsER1* results in increased spikelet numbers. CLV1 is conserved across many crop species, and its knock-out mutants produce multilocular siliques or fruits. In rice, weak alleles of *OsBRI1* confer an erect leaf architecture suited for dense planting.

The primary concern in crop breeding is yield, a complex trait integrating various developmental and physiological processes [25]. At the individual level, yield is typically represented by the size or number of seeds or fruits produced by a single plant, whereas at the population level, it refers to the total amount of seeds or fruits harvested from crops cultivated over a given field area. The role of RLKs in regulating yield-related traits is multifaceted. They affect organ size (e.g., grain, pod, and fruit size), plant architecture (e.g., tillering, leaf angle, and plant height), and meristem size, a trait that ultimately determines the number of flowers, fruit locules, and ovules (Figure 1). Trade-offs among these factors or pleotropic effects are frequently observed in RLK-based breeding studies.

### 3.1. RLKs Regulating Seed or Fruit Size

In Arabidopsis, loss-of-function mutations in LRR-RLK genes such as *ERECTA* (*ER*), *HAIKU2* (*IKU2*), *RLK7*, *TRANSMEMBRANE KINASE 1* (*TMK1*), and the LecRLK gene *LecRLK-VIII.2* have been reported to result in smaller seeds, suggesting their positive roles in regulating grain size [26,27,28,29,30]. In contrast, loss-of-function mutations in the malectin-like RLK gene *FERONIA* (*FER*) lead to larger seeds [31]. Consistently, functional analyses of rice (*Oryza sativa*) FER-like receptors (OsFLRs) demonstrated that knockout mutants of *OsFLR1* or *OsFLR2* also produce larger seeds [32,33]. However, both of the *Osflr1* and *Osflr2* mutants exhibit reduced plant height and lower grain yield per plant compared with the wild type, suggesting a potential pleotropic effect [33]. Other rice RLKs implicated in grain-size regulation include the LRR RLK BRASSINOSTEROID-INSENSITIVE 1 (BRI1)-ASSOCIATED RECEPTOR KINASE 2 (OsBAK2) and the CRINKLY4 RLK designated MINI SEED 2 (OsMIS2/OsCR4), yet their translational application in rice breeding requires further investigation [34,35].

More compelling evidence for the translational potential of RLKs in seed and fruit regulation comes from studies on tomato (*Solanum lycopersicum*) CLAVATA1 (SlCLV1) and peanut (*Arachis hypogaea*) ROOT MERISTEM GROWTH FACTOR 1 (RGF1)-INSENSITIVE 1 (AhRGI1) [36,37]. The LRR-RLK CLV1 detects the CLV3 peptide to trigger downstream signaling that represses expression of *WUSCHEL* (*WUS*), which encodes a transcription factor promoting stem cell accumulation [38,39]. Loss-of-function mutations in *SlCLV1* or other components of the CLV-WUS pathway alter floral organ number, resulting in fruits with three or four locules instead of two, thereby increasing fruit size and weight [36,40].

The LRR-RLK RGI1 is activated by the RGF1 peptide, initiating a downstream MAPK cascade that drives the accumulation of the PLETHORA (PLT1/PLT2) transcription factors essential for root meristem growth [41,42,43]. Through bulked segregation analysis (BSA) sequencing, a recent study identified the *PSW1* (*Pod Size Weight 1*) gene in two peanut accessions with contrasting pod sizes [37]. *PSW1* encodes AhRGI, which also interacts with AhBAK1 [37]. The large-pod allele, designated PSW1^HapII^, contains a 12 bp fragment inserted in the promoter of *PSW1* and a point mutation in its coding region, resulting in a serine-to-isoleucine substitution (S618I). These two mutations markedly enhanced *PSW1* mRNA abundance and its protein binding affinity to AhBAK1, thereby upregulating *AhPLT1* expression and increasing pod size. Through marker-assisted breeding, the authors successfully developed two peanut varieties with increased yield per plant, offering a model for translating fundamental RLK biology into practical breeding applications [37].

### 3.2. RLKs Regulating Seed or Fruit Number

Seed number is determined by both the number of ovules formed within the carpels and the number of flowers produced on the inflorescence [44]. The LRR RLK ER perceives EPIDERMAL PATTERNING FACTOR (EPF) or EPF-LIKE (EPFL) peptides and plays an important role in regulating seed number [45]. In maize (*Zea mays*), although null alleles of *Zmer1* or *Zmer2* result in short ears with a reduced number of seeds, genome-edited weak alleles of *Zmer1* (*Zmer1*^D295N^ and *Zmer1*^A369T^) produce more seeds per ear without severely affecting the ear length [46]. Similarly, rice mutants lacking *OsER1* produced more spikelets per panicle, but seed yield per plant remained comparable to that of wild type because of reduced seed setting and smaller grain size, indicating a trade-off [47]. In contrast, triple mutants of OsER1 ligands, *Osepfl6/7/9*, showed significantly increased spikelet number and grain yield without compromising fertility [48]. Likewise, overexpression of the antagonistic ligand OsEPFL5, which competes with OsEPFL6/7/8/9 for OsER1 binding and suppresses the OsEPFL6/7/9-OsER1 signaling pathway, markedly improved yield without obvious growth penalties [49]. These studies suggest that ER has translational potential, provided that variants with precisely tuned functions can be engineered. Compared with staple food crops, recent studies have also highlighted the translational potential of ER signaling in urban agriculture [50,51]. In Solanaceae fruit crops such as tomato, goldenberry (*Physalis peruviana*), and groundcherry (*Physalis grisea*), CRISPR/Cas9-mediated editing of ER orthologs successfully produced compact, high-yielding dwarf plants suitable for intensive and vertical agriculture [50,51].

CLV1 is another LRR RLK that could be targeted to enhance crop yields. In maize, loss-of-function mutations in *ZmCLV1* have been shown to enlarge the female inflorescence meristem, leading to markedly thicker ears with up to three times the number of kernel rows compared to wild-type plants [52]. Nevertheless, the severe fasciation and disorganized ear structure associated with these strong alleles negatively affect seed yield. Future efforts are still needed to mitigate these pleiotropic effects, potentially through the generation of weak alleles [39]. Alternatively, promoter engineering-mediated knockdown of CLE peptide genes, such as *ZmCLE7* and *ZmFCP1*, successfully enhanced multiple yield-related traits in maize, including kernel row number, ear diameter, ear weight, and grain yield per ear, without apparent adverse side effects [53]. In oilseed crops such as mustard (*Brassica juncea*), two *CLV1* orthologs, *BjA7.CLV1* and *BjB3.CLV1*, have been identified [54,55]. Productive mustard varieties with multi-locular siliques (as opposed to bilocular ones), such as J163-4 and Duoshi, were reported to carry mutations in both *CLV1* genes. In J163-4, an inserted copia-LTR retrotransposable element (RTE1) in the coding region of *BjB3.CLV1* disrupts its transcription, and a 914 bp deletion in the promoter region of *BjA7.CLV1* specifically reduces its expression in the carpel margin meristem (CMM) without affecting its expression in other tissues [54,56]. In Duoshi, *BjA7.CLV1* carries missense mutations resulting in two amino acid substitutions in its coding region and a 702 bp deletion in its promoter region, and *BjB3.CLV1* contains a 4961 bp insertion that disrupts its translation [55,57].

### 3.3. RLKs Regulating Plant Architecture

Ideal plant architecture (IPA, or ideotype) refers to the morphological and structural characteristics of plants that maximize their population yield potential under given environmental conditions [58]. For staple food crops, the IPA generally features a semi-dwarf stature, accompanied by a moderate tiller number and culm diameter to confer lodging resistance and enable dense planting [58].

The LRR-RLK BRI1 functions as the major receptor for brassinosteroids (BRs), a class of steroidal phytohormone recently recognized as a promising driving force for the next Green Revolution [59,60,61,62]. *BRI1* is a potential molecular target for improving crop architecture. A well-known example is the barley (*Hordeum vulgare*) *uzu* cultivars, which are uniquely distributed across East Asia [63]. The semi-dwarfed stature and compact canopy structure of *uzu* barley confer lodging tolerance and pathogen resistance, as well as advantages for dense planting in the field [64,65]. However, *uzu* cultivars also display a heat-sensitive reduction in plant height and fertility when grown in warmer climates, which has limited their global adoption [65,66]. Genetic dissection revealed that *uzu* varieties harbor a specific single nucleotide polymorphism (SNP) resulting in the amino acid substitution H857R in barley HvBRI1 [65]. The mutation is structurally located on the back surface of the BRI1 kinase domain, close to the mutation site of the bri1-301 (G989I) identified in Arabidopsis AtBRI1 [67,68]. Interestingly, *bri1-301* plants also display an allele-specific, thermo-enhanced BR defective phenotype, suggesting a similar underlying molecular mechanism [69,70].

In rice, the weak *Osbri1* allele *d61-7* also exhibit erect leaves suitable for dense planting [71]. Field trials demonstrated that under high-density planting conditions, *d61-7* accumulated greater biomass than wild-type plants, but achieved only comparable grain yield because of undesirable side effects, including reduced fertility and smaller grain size [71]. Recently, a novel strategy was employed to fine-tune BRI1 functionality by generating different upstream open reading frames (uORFs) within the 5′ untranslated region (5′ UTR) of the *BRI1* gene [72]. This approach attenuates mRNA translation, enabling the generation of a series of allelic lines with graded phenotypes [72].

## 4. RLKs in Enhancing Biotic Stress Resistance

**Figure 2:**
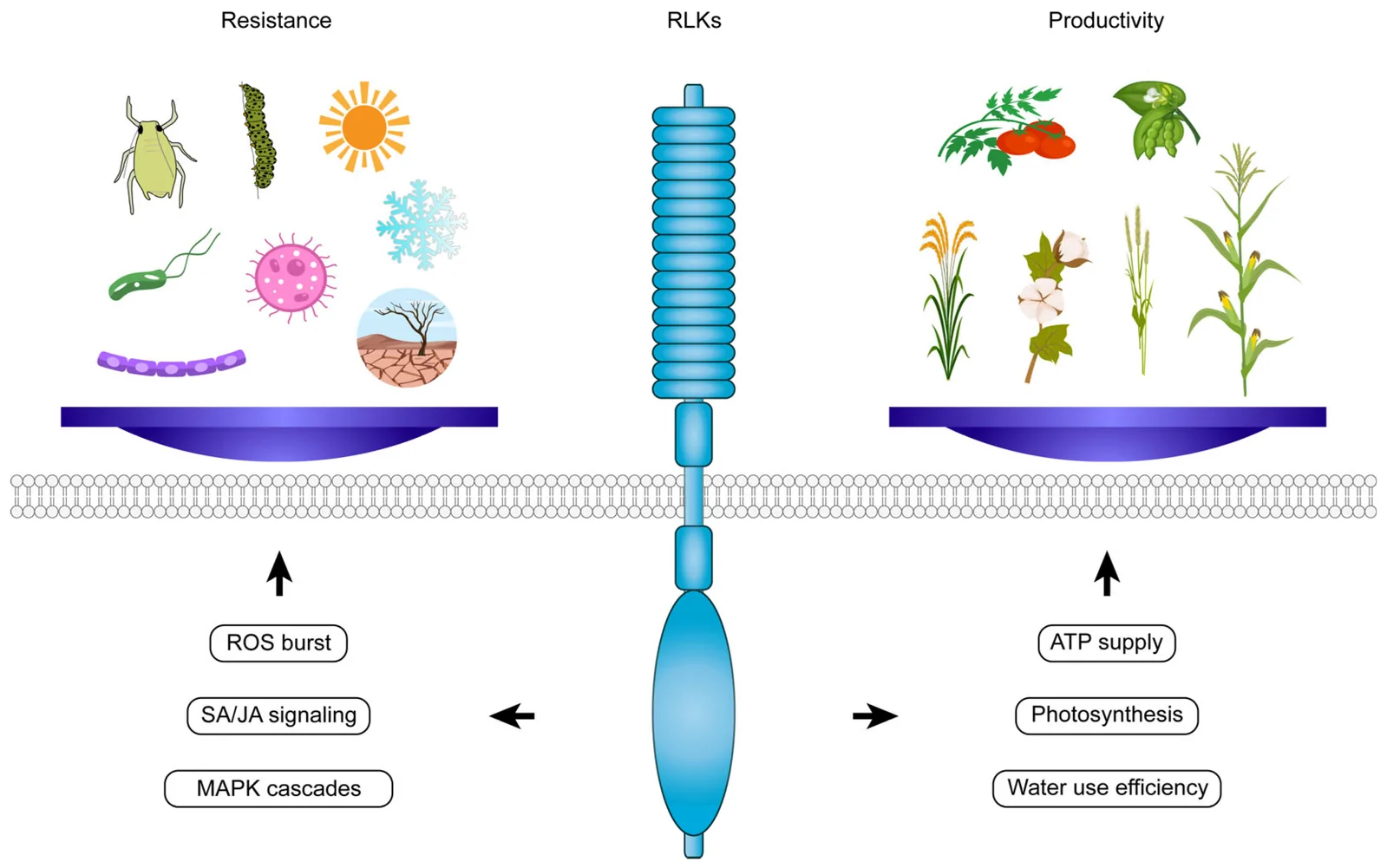
Manipulating RLK signaling to enhance stress resistance without compromising productivity. Studies on several key RLKs have shown that ligand perception activates downstream signaling mediated by the kinase domain, which coordinately regulates plant immunity and productivity. On the one hand, this signaling triggers defense mechanisms such as ROS bursts, SA/JA pathways, and MAPK cascades, leading to the expression of resistance genes. On the other hand, it guarantees productivity by optimizing ATP supply, photosynthesis, and water use efficiency. Here, an LRR RLK is used to represent the RLKs discussed in this review.

Field crops are surrounded by many other organisms, such as phytophagous insects and pathogenic microbes [21,73]. Activities of these organisms often cause biotic stresses that potentially reduce crop yield. Extensive studies revealed that many RLKs are involved in plant defense against biotic stressors [21,73]. Generally, they function as pattern recognition receptors (PRRs) to detect a wide range of exogenous danger signals, including pathogen- or microbe-associated molecular patterns (PAMPs or MAMPs), herbivore-associated molecular patterns (HAMPs), as well as endogenous danger signals such as classic damage-associated molecular patterns (DAMPs) released upon cellular injury, and immunomodulatory peptides or phytocytokines (PCKs) that are processed and/or secreted during infection [74,75,76,77,78,79]. The activation of these RLKs usually activates mitogen-activated protein kinase (MAPK) cascades or triggers hormone signaling pathways involving jasmonic acid (JA) and salicylic acid (SA), leading to the expression of defense-related genes [80,81] (Figure 2).

### 4.1. Resistance to Phytophagous Insects

#### 4.1.1. LRR RLKs

The LRR-RLKs PEPR1 and PEPR2 are PRRs for a class of plant elicitor peptides (PEPs) derived from larger protein precursors known as PROPEPs [82]. In multiple plant species, including Arabidopsis, rice, and maize, the expression of *PROPEP* and *PEPR* genes is strongly upregulated in response to herbivore feeding [83,84,85,86]. Double mutants *pepr1 pepr2* or RNAi knock-down lines exhibit increased susceptibility to herbivory, as larvae feeding on these mutant plants gain significantly more weight or display higher settlement numbers compared to those feeding on wild-type plants [83,86,87]. Therefore, engineered expression of PEP ligands or PEPRs may enhance broad-spectrum insect resistance in various crops, although the potential trade-off in growth and fertility remains to be further explored.

OsLRR-RLK1 is another LRR RLK whose transcription is also upregulated following herbivore attack or treatment with their oral secretions [88]. It positively regulates herbivore-induced activation of trypsin protease inhibitors (TrypPIs) and the accumulation of JA [88]. In rice, TrypPIs serve as anti-digestive proteins that are strongly induced by specific herbivores [89]. Silencing *OsLRR-RLK1* significantly attenuates TrypPI activity and the accumulated JA level, as well as rice resistance to the chewing herbivore striped stem borer (SSB, *Chilo suppressalis*) [88]. Studies in maize indicated that ZmFAC, a close analog of OsLRR-RLK1, may serve as a candidate receptor for fatty acid-amino acid conjugates (FACs) found in herbivore oral secretions [90].

In contrast to OsLRR-RLK1, OsLRR-RLK2 promotes susceptibility to herbivores [91]. Silencing *OsLRR-RLK2* reduces the activity of OsMPK6 and certain WRKYs, lowers endogenous JA levels while increasing SA levels, and enhances rice resistance against the brown planthopper (BPH, *Nilaparvata lugens*) [91]. More importantly, these silencing lines also exhibited more panicles per plant and a higher 1000-grain weight compared to wild-type plants, resulting in increased grain yield [91]. Another OsLRR-RLK, OsRLK7-1, was recently reported to be induced by DAMPs and also responds to BPH [92]. Its knockout mutant gives strong BPH resistance but causes serious growth and reproduction problems, showing the typical defense-growth trade-off [92]. Strategies that uncouple defense from growth penalty, such as tissue-specific or inducible expression, may solve this issue.

The peptide hormone systemin plays key roles in mediating responses to insect herbivory [93]. A recent study revealed that two tomato LRR-RLKs, SlSYR1 and SlSYR2, function as systemin receptors to activate defense responses against insect herbivores [94]. Larvae feeding on a tomato IL3-3 introgression line, which lacks SlSYR1/SlSYR2, exhibited improved performance and gained significantly more mass compared to those feeding on wild-type plants [94].

#### 4.1.2. LecRLKs

LecRLKs constitute another major subfamily of RLKs which is characterized by the extracellular lectin domain. The subfamily can be divided into three groups (G-type, C-type, and L-type) based on the structural features of the lectin domain [95]. LecRLKs also play important roles in herbivore resistance. In rice, *Bph3* is a brown planthopper (BPH)-resistant locus identified 50 years ago in a rice cultivar Rathu Heenati [96]. It has been demonstrated previously that *Bph3* consists of a cluster of three *LecRLK* (*OsLecRK1*–*OsLecRK3*) genes that function additively to confer broad-spectrum and durable resistance to both BPH and the white back planthopper (WBPH) [97]. Sequence analysis revealed that resistant rice varieties possess identical amino acid sequences for these three genes, while susceptible varieties harbor deleterious polymorphisms [97]. Marker-assisted breeding by introducing the *Bph3* locus from Rathu Heenati into the susceptible commercial japonica variety Ningjing 3 significantly enhanced its durable resistance to both BPH and WBPH [97].

### 4.2. Resistance to Pathogenic Microbes

#### 4.2.1. LRR RLKs

The LRR-RLK FLS2 (Flagellin-Sensing 2) is a well-characterized plant PRR that recognizes bacterial flagellin upon pathogen attack and, together with the co-receptor BAK1, initiates the pattern-triggered immune (PTI) responses [98,99]. However, some pathogens carrying flagellin variations may evade this detection mechanism due to reduced binding affinity for FLS2 or impaired BAK1 recruitment [100]. Recent studies revealed that the narrow perception range of various ligands for a given receptor can be broadened by rational engineering key residues on the LRR concave surface of FLS2, which interact with the flg22 (N-terminus of flagellin) and the SERK co-receptor [101,102,103]. For example, a designed mutation of Q600H and S603A in soybean (*Glycine max*) GmFLS2a enhanced the direct FLS2-BAK1 interaction and, thereby improving recognition of flg22Rso, a flg22 variant that retained the ability to interact with FLS2 but impeded BAK1 recruitment [101]. These studies provide a framework for the rational engineering of RLKs to improve pathogen resistance, as well as potentially other agronomic traits [101,102,103].

In rice, the LRR-RLK OsSRF3 (STRUBBELIG-RECEPTOR FAMILY 3) was reported to play a key role in resistance against multiple pathogens. OsSRF3 interacts with and is phosphorylated by OsBAK1, thereby activating pathogen-triggered immunity, including the MAPK cascade, ROS burst, callose deposition, and expression of defense-related genes. Notably, overexpression of *OsSRF3* enhances resistance without negatively impacting plant growth or yield [104]. A recent study revealed that an LRR-RLK OsRLK5 detects the PCK OsIMP1 to confer resistance against both bacterial and fungal diseases in rice [105]. OsIMP1 is transcriptionally induced by PAMPs such as flg22 and chitin and directly binds to OsRLK5 as well as its paralog, OsRLK5-L. Ligand perception by OsRLK5 enhances its interaction with the co-receptor OsSERK2, thereby facilitating trans-phosphorylation required for downstream signaling. Notably, *OsRLK5*-overexpressing lines exhibited significantly enhanced resistance to *Xanthomonas oryzae pv. oryzae* (bacterial blight) and *Magnaporthe oryzae* (rice blast), without any apparent growth trade-off or yield penalty, presenting a promising molecular target for engineering disease-resistant rice germplasm [105].

In wheat, similar to the OsIMP1, a family of small, cysteine-rich peptides known as Delta-like PCKs (DEPs) was found to be rapidly induced by PAMPs and to confer multi-pathogen resistance in wheat. DEP2 are recognized by the LRR-RLK DEPR1, which recruits the co-receptor SERK2 upon ligand binding. Although exogenous application of synthetic DEP2 peptide and transgenic overexpression of *preDEP2* both significantly enhance wheat resistance to multiple pathogens, genetic disruption of DEP signaling via DEPR1 mutation unexpectedly does not lead to increased susceptibility, suggesting the presence of functional redundancy or the activation of compensatory immune pathways [106].

#### 4.2.2. LecRLKs

Recent studies demonstrated a huge potential of LecRLKs in improving crop pathogen resistance. In rice, an L-type LecRLK designated OsLIKE1 was shown to play a critical role in resistance against *Magnaporthe oryzae* [107]. In resistant rice varieties, specific mutations within the *OsLIKE1* promoter region significantly enhance its light-induced expression, thereby promoting the autophosphorylation of OsLIKE1 and activating its immunity against the blast fungus [107]. Importantly, compared to wild-type ZH11 plants, *OsLIKE1-overexpressing (OE)* lines maintained comparable plant height and grain yield upon *M. oryzae* infection [107]. Another LecRLK, OsBDR1, was recently identified as a negative regulator of rice blast resistance. Upon *Magnaporthe oryzae* infection, BDR1 was rapidly induced and directly interacts with and phosphorylates MPK3. The BDR1-MPK3 module suppresses JA signaling and the biosynthesis of antifungal terpenoids, including the monoterpene linalool and the sesquiterpene caryophyllene. Importantly, knockout of *OsBDR1* confers enhanced resistance to blast fungus without significant agronomic penalties, offering a promising target for gene-editing breeding [108].

OsSBRR1 is a G-type LecRLK that confers rice resistance to sheath blight (ShB), a disease caused by the necrotrophic fungus *Rhizoctonia solani*. A recent genome-wide association study (GWAS) revealed that in ShB-resistant cultivars, a 256 bp insertion in the *OsSBRR1* promoter is essential for its strong transcriptional induction upon *R. solani* infection. SBRR1 is able to rapidly upregulate the expression of several chitinases capable of degrading fungal cell walls. Notably, marker-assisted introgression of this elite *SBRR1-R* allele into commercial *japonica* cultivars significantly enhanced ShB resistance and mitigated approximately 10% of grain yield loss under severe disease pressure, without incurring adverse agronomic effects, thereby highlighting its strong potential for breeding applications [109].

In wheat, Septoria tritici blotch (STB) is a major disease caused by the Dothideomycete fungus *Zymoseptoria tritici*. A recent GWAS involving 300 wheat landraces identified a G-type LecRLK, designated *Stb15*, which confers resistance to STB, as confirmed through both EMS mutagenesis and stable gene transformation. The functional *Stb15* allele contains an intronless open reading frame, while the susceptible allele in cv. Chinese Spring includes four introns [110]. Wheat powdery mildew, another disease caused by *Blumeria graminis* f. sp. *tritici* (*Bgt*), can cause serious yield loss. An L-type LecRLK, designated LecRK-V, was cloned from *Haynaldia villosa*, a diploid wheat relative. This gene was responsible for its high resistance to powdery mildew. The expression of *LecRK-V* was rapidly upregulated upon *Bgt* inoculation or chitin treatment. Stable transformation of *LecRK-V* into the susceptible wheat variety Yangmai158 significantly enhanced its resistance to multiple *Bgt* isolates, indicating its involvement in regulating broad-spectrum resistance [111].

In maize, GWAS also identified a G-type LecRLK, ZmLecRK1, as a resistance protein against *Pythium aphanidermatum*, one of the causal pathogens of stalk rot. In resistant maize inbred lines, a naturally occurring S404A mutation in ZmLecRK1 ED strengthens its binding affinity to the co-receptor ZmBAK1, thereby enhancing the formation of active complexes for defense response [112].

#### 4.2.3. CRKs

CRKs belong to an RLK subfamily containing one to four DUF26 (domain of unknown function 26) in their ED, each characterized by several conserved cysteine residues arranged as a C-X8-C-X2-C pattern [113]. CRKs are proved to be involved in the perception of extracellular abiotic and biotic signals [113].

In wheat, it was reported that *Stb16q*, an initially identified quantitative locus conferring resistance to STB, encodes a CRK that significantly reduces the penetration and intercellular growth of the pathogen during infection [114,115]. Sequence analysis of *Stb16q* across diverse wheat accessions revealed that this gene was recently introduced into modern cultivars through synthetic wheat breeding [115]. Additionally, another CRK, TaCRK6, was identified and functionally characterized in the wheat cultivar Xiaoyan 6 (XY6), which exhibits non-race-specific and durable high-temperature all-stage (HTAS) resistance to stripe rust (*Puccinia striiformis* f. sp. *tritici*, *Pst*). The expression of TaCRK6 is synergistically induced by *Pst* infection and elevated temperature. Silencing *TaCRK6* in XY6 compromises HTAS resistance, whereas its overexpression in the susceptible cultivar Fielder enhances resistance without agronomic penalties, making it a promising target for breeding climate-resilient wheat with durable stripe rust resistance [116].

#### 4.2.4. WAKs and WAKLs

WAKs and WAKLs (WAK-like) constitute an RLK subfamily containing an epidermal growth factor (EGF)-like domain and/or a calcium-binding EGF domain in their ED. These RLKs are implicated in cell wall signal sensing. They are responsible for transmitting extracellular signals to the cytoplasm, thereby regulating plant growth, development, and stress responses [117,118].

In wheat, molecular characterization of an *Stb6* gene, which provides gene-for-gene resistance to the fungal pathogen *Zymoseptoria tritici*, the causal agent of STB disease, revealed that it encodes a WAKL designated TaWAKL4. Haplotype analysis indicated that a single predominant resistance haplotype is broadly conserved in both bread wheat and its tetraploid progenitors, suggesting that *Stb6* was introduced early during wheat domestication [119].

In maize, it was reported that several WAKs and WAKLs were required for the resistance to diseases such as northern corn leaf blight (NCLB), head smut, and gray leaf spot (GLS) [120,121,122]. NCLB is a major foliar disease of maize. The *Htn1* locus confers quantitative and NCLB resistance by delaying the onset of lesion formation, therefore representing an important source of genetic resistance. *Htn1* was originally introduced from a Mexican landrace into modern maize breeding lines in the 1970s. Genetic mapping and molecular characterization demonstrated that *Htn1* encodes ZmWAK-RLK1, which contains a non-arginine-aspartate (non-RD) kinase domain typically found in plant innate immune receptors. Sequence comparison showed that the extracellular domain of ZmWAK-RLK1 is highly diverse between different maize genotypes [121].

GLS is another maize foliar disease, and its resistance is inherited as a typical quantitative trait with many loci identified [123]. *qRgls1* is one of such loci identified through quantitative trait locus (QTL) mapping, which enhances resistance percentages by 19.70–61.28% [124]. A recent study identified *ZmWAKL* as the causal gene underlying the resistance of the *qRgls1* locus against GLS. Similar to ZmWAK-RLK1, the ZmWAKL protein exhibits a markedly different extracellular domain between resistant and susceptible maize inbred lines. Upon pathogen infection, the resistant protein ZmWAKL^Y^ rapidly self-associates and interacts with the LRR-RLK coreceptor designated ZmWIK. The complex then phosphorylates an RLCK ZmBLK1, which in turn phosphorylates the NADPH oxidase ZmRBOH4, triggering a reactive oxygen species (ROS) burst that confers quantitative resistance [122].

*qHSR1* is a major QTL conferring resistance to head smut, another devastating maize disease that causes severe yield losses worldwide [125]. Introduction of *qHSR1* into susceptible lines significantly reduces disease incidence across years, demonstrating its great potential in breeding [126]. Fine mapping revealed that *qHSR1* encodes a ZmWAK, which contains an extracellular galacturonan-binding (GUB) domain and a calcium-binding EGF domain, as well as a cytoplasmic non-RD serine/threonine kinase domain. Mechanistically, ZmWAK reduces disease incidence by specifically arresting the biotrophic growth of the pathogen in the mesocotyl, the key tissue that the fungus must traverse to reach the shoot meristem. Resistance was strongly associated with *ZmWAK* expression levels in the mesocotyl, emphasizing the importance of spatiotemporally optimized defense in maize [120].

## 5. RLKs in Enhancing Abiotic Stress Resistance

In addition to biotic stresses, field crops also face challenges from abiotic stresses such as low or high temperature, drought, and high salinity, leading to yield reductions [127]. Previous studies shed light on the role of RLKs in abiotic stress responses and the potential mechanisms underlying RLK-mediated abiotic stress tolerance [128,129,130].

### 5.1. Heat Tolerance

Studies in Arabidopsis demonstrate that ERf receptors function redundantly and synergistically to modulate stomatal density, spacing, and lineage proliferation. Dysfunction of the ER leads to increased stomatal density and conductance, accompanied by reduced transpiration efficiency and water use efficiency (WUE), ultimately impairing heat tolerance [131,132]. In contrast, ER overexpression improves heat tolerance. Field validation across multiple locations and seasons showed that transgenic tomato and rice lines overexpressing *ER* survive heat stress in both greenhouses and open summer fields and produce larger leaves, more inflorescences, higher seed set, greater shoot dry weight, and increased biomass and yield compared with controls [131].

A recent study in Arabidopsis demonstrated that FER acts as a membrane-anchored thermal switch that orchestrates plant heat acclimation through dynamic nanocluster assembly [133]. Under a moderate heat condition (37 °C), a peptide ligand RALF34 activates FER, which is recruited to sterol-rich plasma membrane nanodomains. There, FER nucleates signaling nanoclusters enriched in components such as UGT80A2, REMORINs, and BSKs, stabilizing liquid-ordered membrane phases and activating the HSF-HSP cascade to enhance thermotolerance. This process depends on heat-induced sterol glycosylation and anionic lipid binding by the FER juxtamembrane domain. Under an extreme heat condition (>40 °C), nanoclusters rapidly disassemble due to actin cytoskeleton collapse, preventing maladaptive signaling [133].

### 5.2. Cold Tolerance

Cold stress not only affects plant growth and development, but also influences plant productivity [134]. Cold-induced pollen abortion and reproductive failure are key causes of yield losses in many crops [135].

In tomato, a recent study revealed that an LRR-RLK SlRGFR6 plays a crucial role in mediating SlRGF peptide signaling, thereby safeguarding pollen development and yield under chilling stress [136]. RGF peptides, including SlRGF9 and SlRGF10, are specifically upregulated in the anther tapetum upon cold exposure. They bind to SlRGFR6 and the co-receptors SlSERK1/3A/3B, triggering calcium influx through SlCNGC16/18 channels. The resulting calcium signaling counteracts cold-delayed programmed cell death of the tapetum, ensuring its timely degradation to support microspore development. Loss-of-function mutants of this pathway exhibit severe pollen abortion under cold stress, while moderate overexpression of *SlRGF9* or *SlRGF10* in tomato prevents up to 52% of chilling-induced yield loss. This mechanism is conserved in rice, as up-regulation of the homologous *OsRGF10* similarly enhances cold resilience and recovers up to 18.3% of grain yield loss, providing a promising target for engineering climate-resilient crops [136].

In rice, it was also reported that an LRR-RLK, designated OsCTB4a, confers cold tolerance at the booting stage in rice [137]. CTB4a interacts with AtpB, the beta subunit of ATP synthase, resulting in increased ATP synthesis and photosynthetic rate [137]. This provides sufficient energy for pollen development and grain filling under low-temperature conditions. Under cold field conditions, a near-isogenic line NIL1913 and *CTB4a* overexpression lines exhibited superior performance in terms of filled grain number, thousand-kernel weight, and yield per plant [137]. QTL mapping and natural variation analysis indicated that SNPs in the promoter region of *OsCTB4a*, particularly at positions -2536, -2511, and -1930, result in stronger cold-inducible expression of *OsCTB4a* in the cold-tolerant haplotype Hap-KMXBG compared to the cold-sensitive haplotype Hap-Towada [137]. Geographic analysis demonstrated that Hap-KMXBG is predominantly distributed in temperate *Japonica* rice varieties from high-latitude regions such as Northeast China, Japan, and Korea, as well as high-altitude regions like the Yunnan–Guizhou Plateau, and exhibits clear signatures of artificial selection for cold adaptation [137]. This example illustrates that RLK-mediated cold tolerance can be engineered without frequently incurring trade-offs.

### 5.3. Drought Tolerance

RLKs play important roles in optimizing plant responses to drought stress, and are considered as potential targets for crop improvement [138].

In wheat, the LRR RLK TaCEPRL has been reported to confer drought tolerance [139]. Compared with wild-type plants, *TaCEPRL* knockout mutants showed no obvious phenotypic differences at various growth stages under normal conditions [139]. However, they exhibited significantly enhanced drought tolerance, reflected in higher survival rates, greater thousand-kernel weight, increased chlorophyll content, and improved water use efficiency [139]. Similarly, another LRR RLK, TaCLV1, also contributes to drought tolerance in wheat [140]. Knockout of *TaCLV1* resulted in a reduced seedling survival rate under drought stress, whereas overexpression of its ligand, *TaCLE24b*, increased seedling survival rates, along with higher chlorophyll content in flag leaves and greater thousand-kernel weight under water-deficient conditions, but not under normal conditions [140].

In rice, an LRR RLK OsLRK2, which is phylogenetically closely related to Arabidopsis PSKR1, also plays a role in drought tolerance [141]. *OsLRK2* is constitutively expressed across multiple rice tissues and is significantly induced under drought stress [141]. Overexpression of *OsLRK2* enhances drought tolerance by delaying leaf rolling, increasing survival rates, and improving recovery growth following drought treatment [141]. These benefits are attributed to the development of a larger root system, characterized by more lateral roots and higher root activity. Moreover, overexpression lines produce more panicles, leading to a significant increase in grain yield per plant [141]. Overall, OsLRK2 promotes cell proliferation and the branching of tillers and lateral roots, thereby improving both drought tolerance and yield-related traits in rice [141].

Furthermore, a recent study in rice identified OsCRK14, a cysteine-rich receptor-like kinase, as a key positive regulator of drought resistance [142]. *OsCRK14* is transcriptionally upregulated under drought stress, and its overexpression in rice lines resulted in significantly higher survival rates compared to wild-type ZH11 [142,143]. OsCRK14 phosphorylates OsRLCK57 under drought stress, leading to activation of the MAPK cascade OsMKKK10-OsMKK4-OsMPK6. Activated OsMPK6 then directly phosphorylates the abscisic acid-responsive transcription factor OsbZIP66, enhancing its stability and promoting the expression of drought-responsive genes. Natural variations in the *OsCRK14* promoter region influence its transcript levels by altering OsMYB72 binding affinities, which are correlated with differences in drought resistance among rice varieties [143].

### 5.4. Salt Tolerance

In Arabidopsis, it was reported that FER orchestrates salt tolerance through maintaining cell-wall integrity and inhibiting photorespiration [144,145,146,147]. Another malectin-like RLK, SIMP1, is critical for plant salt tolerance by ensuring efficient proteasomal clearance of aberrant proteins resulting from endoplasmic reticulum stress induced by salinity [148]. Lectin RLKs are also essential for plant salt response. Studies in rice revealed that a lectin RLK, designated OsSIT1, is rapidly phosphorylated and activated by salinity to promote ROS-induced growth inhibition [149,150]. Its knockout mutant showed substantially improved growth under high-salt conditions but a severely reduced grain yield due to effects on growth and development, thereby establishing a trade-off between salt tolerance and normal growth [149]. Interestingly, overproduction of Hap2-OsSIT1, a natural variant of OsSIT1 with lower kinase activity, conferred improved seedling growth under salt stress without obvious yield penalties [151]. Similarly, overexpressing an *OsSIT1* homolog *OsSIT2* in pigeon pea (*Cajanus cajan*) resulted in 10.34-fold increase in seed yield per plant and a 4–5-fold increase in harvest index relative to stressed wild-type plants, suggesting a significantly enhanced salinity stress tolerance [152]. Moreover, transgenic soybean plants overexpressing another *LecRLK* also gave rise to significantly higher plant height and yield than those of WT under salt stress [153].

## 6. Challenges and Future Perspectives

Over the past two decades, a substantial body of literature has documented the roles of RLKs in regulating diverse biological processes [79]. Notably, some RLK-mediated pathways are closely linked to key agronomic traits, making them promising molecular targets for crop improvement. As summarized above, RLKs can be engineered to (1) enhance crop yield by increasing grain number, enlarging fruits, or optimizing plant architecture for dense planting, (2) bolster resistance to biotic stresses, including insect herbivores and bacterial/fungal pathogens, and (3) improve tolerance to abiotic stresses such as heat, cold, drought, and salinity (Table 1).

Despite these advancements, substantial challenges remain. First, some RLKs exhibit pleiotropic effects. Simply knocking out or overexpressing an *RLK* gene may enhance a target trait, it also produces undesirable impacts on other agronomic traits. Several strategies can be applied to mitigate or uncouple the multifaceted functions of a given RLK: (1) introducing known weak or beneficial mutations through base editing or prime editing, (2) fine-tuning tissue-specific expression via promoter engineering approaches such as uORF editing, and (3) designing novel mutations using artificial intelligence-based methods, including protein structure prediction, molecular docking, and molecular dynamics simulations [5,154,155]. Second, some RLKs exhibit functional redundancy with other members of their subfamily, a phenomenon especially evident in polyploid crops. As a result, single-gene mutations often fail to produce the expected phenotypic changes. Nevertheless, this limitation can be overcome by advances in multiplex CRISPR editing and haplotype-based breeding strategies [155,156,157]. Third, the evolutionary divergence of certain RLKs across plant species (e.g., between monocots and dicots) may render agronomically valuable mutations identified in one species of little or no utility in another. Moreover, even within a single crop species, such a mutation may confer benefits only under specific environmental conditions while proving ineffective under others. Therefore, in translational applications, the functional requirements of RLK alleles should be carefully evaluated across different species and environmental conditions.

Individual RLKs usually perceive specific signals and modulate distinct downstream pathways. The simultaneous design and editing of multiple RLKs, rather than manipulation of a single receptor, may offers a promising route to combine yield potential, abiotic stress tolerance, and disease resistance within a single elite genetic background. With the accelerating pace of functional genomic studies in crops and the continued development of such multiplex strategies, RLKs represent promising targets for molecular breeding toward sustainable, high-yielding, and resilient agriculture.

## Figures and Tables

**Table 1:** Featured RLKs and mutations potentially useful in crop improvement.

Subfamily	Name	Species	Mutant	Breeding Strategy	Trait	Side-Effect	Ref.
LRR RLKs	BRI1	Rice	*d61-7*	Natural variation	Compact architecture	Yes	[71]
		Rice	*uORF*	uORF editing	Compact architecture	Yes	[72]
		Barley	*uzu*	Natural variation	Compact architecture	Yes	[63,64,65,66]
	ERECTA	Rice	*Oser1*	Knock out	Increased spikelet number	Yes	[47]
		Maize	*Zmer ^D295N^* *Zmer1* ^A369T^	Crispr-editing	Increased kernel row number	Yes	[46]
		Tomato	*Sler1*	Knock out	Compact architecture	No	[51]
		Tomato	*SlER-OE*	Overexpression	enhanced heat tolerance	No	[131]
		Rice	*OsER-OE*	Overexpression	enhanced heat tolerance	No	[131]
	CLV1	Maize	*td1*	Knock out	Increased kernel row number	Yes	[52]
		Mustard	*mc1/2*; *Bjln1/2*	Natural variation	Increased seed number	No	[54,55,56,57]
		Tomato	*fab*	Natural variation	Enlarged fruits	No	[36,40]
	RGI1	Peanut	*PSW1^HapII^*	Natural variation	Increased seed size	No	[37]
		Tomato	*Slrgfr6-ko*	Knock out	decreased cold tolerance	No	[136]
	OsRLK2	Rice	*ir-lrr2*	Knock down	Resistant to BPH	No	[91]
	OsRLK7-1	Rice	*ko-rlk7*	Knock out	Resistant to BPH	Yes	[92]
	GmFLS2	Soybean	Q600H/S603A	Artificial design	Broadened ligand spectrum	NS	[101]
	OsSRF3	Rice	*OsSRF3-OE*	Overexpression	Resistant to multiple pathogens	No	[104]
	OsRLK5	Rice	*OsRLK5-OE*	Overexpression	Resistant to bacterial blight and rice blast	No	[105]
	OsCTB4a	Rice	*OsCTB4a^R^*	Natural haplotype	Enhanced cold tolerance	No	[137]
	TaCEPRL	Wheat	*Taceprl-ko*	Knock out	Enhanced drought tolerance	No	[139]
	OsLRK2	Rice	*OsLRK2-OE*	Overexpression	Enhanced drought tolerance	No	[141]
LecRLKs	LecRLK1/2/3	Rice	*Bph3^R^*	Natural haplotype	Resistant to BPH and WBPH	No	[97]
	TaLecRLK	Wheat	*Stb^R^*	Natural haplotype	Resistant to Septoria tritici blotch	No	[110]
	OsLIKE1	Rice	*OsLIKE1-OE*	Overexpression	Resistant to rice blast	No	[107]
	OsBDR1	Rice	*Osbdr1-ko*	knock out	Resistant to rice blast	No	[108]
	OsSBRR1	Rice	*OsSBRR1^R^*	Natural haplotype	Resistant to sheath blight	No	[109]
	TaLecRLK-V	Wheat	*TaLecRLK-V-OE*	Overexpression	Resistant to powdery mildew	No	[111]
	ZmLecRK1	Maize	*ZmLecRK1^S404A^*	Natural variation	Resistant to stalk rot	No	[112]
	OsSIT1	Rice	*OsSIT1^R^*	Natural haplotype	Enhanced salt tolerance	No	[151]
CRKs	TaCRK	Wheat	*Stb16q^R^*	Natural haplotype	Resistant to Septoria tritici blotch	No	[115]
	TaCRK6	Wheat	*TaCRK6-OE*	Overexpression	Resistant to stripe rust	No	[116]
	OsCRK14	Rice	*OsCRK14-OE*	Overexpression	Enhanced drought tolerance	Yes	[142]
WAKs	TaWAKL4	Wheat	*Stb6^R^*	Natural haplotype	Resistant to Septoria tritici blotch	No	[119]
	ZmWAK-RLK1	Maize	*Htn1^R^*	Natural haplotype	Resistant to northern corn leaf blight	No	[121]
	ZmWAKL	Maize	*qRgls1^R^*	Natural haplotype	Resistant to gray leaf spot	No	[124]
	ZmWAK	Maize	*qHSR1^R^*	Natural haplotype	Resistant to head smut	No	[126]

NS, not shown.

## Data Availability

No new data were created or analyzed in this study. Data sharing is not applicable to this article.
